# Biochars derived from bamboo and rice straw for sorption of basic red dyes

**DOI:** 10.1371/journal.pone.0254637

**Published:** 2021-07-14

**Authors:** Ebenezer Ampofo Sackey, Yali Song, Ya Yu, Haifeng Zhuang

**Affiliations:** 1 School of Civil Engineering and Architecture, Zhejiang University of Science and Technology, Hangzhou, Zhejiang, China; 2 Key Laboratory of Recycling and Eco-Treatment of Waste Biomass of Zhejiang Province, Zhejiang University of Science and Technology, Hangzhou, Zhejiang, China; King Saud University, SAUDI ARABIA

## Abstract

The primary purpose of this study is to eliminate Basic Red 46 dye from aqueous solutions utilizing batch experiments by adsorption on biochars prepared from bamboo and rice straw biomass. Biochars prepared from bamboo (B), and rice straw (R) was pyrolyzed at 500°C (B500 and R500). Scanning Electron Microscopy (SEM), Fourier Transform Infrared (FTIR) Spectroscopy, X-ray Diffraction (XRD), and surface area and porosity analyzers were used to characterize the B500 and R500 samples. The characterization results indicated that the biochars possessed an amorphous porous structure with many functional groups consisting primarily of silicates. The adsorption rate of BR46 was evaluated using two kinetic models (pseudo-first-order and pseudo-second-order), and the results indicated that the pseudo-second-order model fitted to the experimental data well (R^2^>0.99). Nearly 24 h was sufficient to achieve equilibrium with the dye adsorption for the two biochars. R500 had a greater adsorption efficiency than B500. As pH levels increased, the dye’s adsorption capability increased as well. The Langmuir and Freundlich isotherm models were used to investigate the equilibrium behavior of BR46 adsorption, and the equilibrium data fitted well with the Langmuir model (R^2^>0.99) compared to the Freundlich model (R^2^>0.89). The maximum adsorption capacities of BR46 are 9.06 mg/g for B500 and 22.12 mg/g for R500, respectively. Additionally, adsorption capacity increased as temperature increased, indicating that adsorption is favored at higher temperatures. The electrostatic interaction is shown to be the dominant mechanism of BR46 adsorption, and BR46 acts as an electron-acceptor, contributing to n-π EDA (Electron Donor-Acceptor) interaction. Thermodynamic parameters for the dye-adsorbent system revealed that the adsorption process is spontaneous and feasible. The values of the adsorption coefficient (K_d_) were on the order of 10^2^−10^3^. K_d_ of R500 was greater than that of B500, indicating that R500 had a greater adsorption capacity. The results showed that R500 could be used as a low-cost alternative adsorbent for removing BR46 from effluents.

## Introduction

Textile, paper, leather, pulp, cosmetics, and dye production sectors produce highly colored effluents due to the presence of organic chemicals known as dyes [1]. Textile industries immensely process dyes, chemicals, and water during fabric manufacture. This produces a large amount of wastewater with robust persistent color and a high BOD loading, making it esthetically and environmentally objectionable [2]. However, synthetic dyes’ use in various industries has expanded dramatically over the years due to their ease of application and long-lasting dying effect [3]. Dispersal of dyes into bodies of water results in colored water, which is a noticeable public health hazard [1]. These distributed dye molecules prevent sunlight from reaching the majority of the affected water body, decreasing the amount of dissolved oxygen (DO) in the water. Dyes may also raise the polluted water body’s biochemical oxygen demand (BOD) [4]. The toxicity level of a dye is critical because of the different effects on the ecosystem and living creatures [5].

Azo dyes are mainly used in the textile industries, which have become harmful to the environment [6]. While certain dyes are not known to be hazardous in the short term, others, notably azo dyes, are. To be precise, azo dyes generate aromatic amines, which are very poisonous, carcinogenic, and even combustible when the azo group is reductively cleaved [7]. BR46 dye is an industrial dye used as a model compound for Azo dyes, identified as one of the most problematic dyes in industrial effluents, and can be potentially life-threatening for living organisms [8]. Furthermore, the most frequent carcinogens, such as benzidine, are found in most dyes that must be treated before release into the environment [9]. This is a strong indication that wastewater treatment to remove Azo dyes such as BR46 is urgent and needs to be solved [10].

Several approaches and procedures for removing dyes from industrial effluents and other water systems have been developed to control the detrimental effects on living organisms [11]. Separation techniques have been used to remove various pollutants from wastewater, including microbial degradation, advanced oxidation, membrane separation, reverse osmosis, coagulation, ion exchange, solvent extraction, filtration, and adsorption [12–14]. Advanced oxidation techniques are very effective yet expensive because of the necessary high energy [15].

Among these separation methods, adsorption is a straightforward, cost-effective, and environmentally friendly form of wastewater treatment, and the manufacturing and research of novel adsorbents has generated considerable interest [16]. Commercial adsorbents such as silica gel, zeolite, resins, graphene, chitosan, activated carbon, lignin, clay, alumina, and charcoal have been used to remove harmful contaminants from wastewater [10, 11, 17–19].

Numerous researchers have concentrated their efforts in recent years on developing less expensive adsorbents for dye removal, including wheat straw, cabbage waste, peanut husk, coconut waste, yellow mombin fruit stones, banana peel, cashew nutshell, canola stalk, teak leaf, and mango seed husks [20–27]. These agricultural by-products are rapidly being used due to their low ash content, good mechanical characteristics, few processing needs, outstanding adsorption potential, accessibility, and regenerative ability [28]. Moreover, the development of adsorbents from various biomass wastes as a replacement of commercial activated carbons further adds to the cost-effectiveness of the process [29].

In a wide range of organic and inorganic pollutants dissolved in aqueous mediums or gaseous environments, activated carbon has been demonstrated to be an effective adsorbent in removing contaminants [30]. Activated carbon is also recognized for the pores, vast porous surface area, thermostabilities, and low acid/base reactivity as very efficient adsorbents [31]. These features enable the treatment of wastewater (heavy metals, dyes, pesticides, etc.), metal recoveries, and catalysis as a means of cleansing material in the areas of pollution control, solvent recovery, the chemical and pharmaceutical industry [32].

Research into the usage of biochar produced from biomass pyrolysis is progressing even if activated carbon is still the most commonly employed adsorbent [33]. This is possible because this type of material has several advantages, such as the fact that they are ecologically safe and financially lucrative [34]. In addition, hydrothermal carbonization of biomass, as an alternative to pyrolysis, results in hydrochar [35]. However, hydrochar has a lower porosity and surface area than biochar, lowering its adsorption efficiency [36].

Biochar is a practical, naturally friendly, and cost-effective adsorbent used to treat and extract various inorganic and organic contaminants from water [37]. Low-cost biochar with exceptional textural properties (high total pore volume, large specific surface area, and excellent chemical stability) have recently been employed as adsorbents to remove dyes and heavy metals from aqueous solutions [38]. The process of biochar adsorption mainly depends on pyrolysis conditions, surface functional properties, feedstock forms, and biochar porosity [39]. Several studies have reported on the adsorption of inorganic and organic pollutants from aqueous solutions using biochars [21, 40]. They have investigated the impact of several factors such as pyrolysis temperature and solution pH on the adsorption process [41]. However, few involved removing BR46 using biochar [4, 42]. A. Ahmad et al. reported that biochar derived from cow dung pyrolyzed at 500°C was efficient for removing the dye in wastewater [43].

Bamboos are a rapidly growing plant biomass that thrives in subtropical, tropical, and temperate climate zones. They have been employed as valuable raw resources and construction materials and have significant cultural and economic value in China [44]. Each year, a substantial amount of bamboo waste is generated, with a high concentration of lignocelluloses as a possible resource for char manufacturing [3]. In China, rice is the primary carbohydrate source. According to the United Nations’ Food and Agriculture Organization (FAO), global rice production totaled approximately 755 million tons in 2019 [45]. Traditionally, rice straw has been regarded as a waste, and as such, has been left or burned with no benefit. It will be proper to utilize this biomass waste by making biochar to improve water quality before its release into the environment. To our knowledge, little study has been conducted on the removal of BR46 dye from wastewater using biochar. As a result, this research examines the possible applicability of bamboo and rice straw-derived biochar for the removal of BR46 dye from aqueous solution by batch mode adsorption experiments.

The potential and feasibility of employing bamboo and rice straw-derived biochar as a sorbent for extracting basic dye from wastewater are investigated in this study, and the most appropriate biochar for this application. The adsorption capacities of the derived biochars were examined using BR46 as a model dye, and the dye adsorption mechanism is explained. Additionally, two primary models are utilized to illustrate adsorption kinetics: pseudo-first-order and pseudo-second-order, while two equations, Langmuir and Freundlich, are employed to characterize the equilibrium data. Additionally, thermodynamic parameters and adsorption affinity are determined for BR46 adsorption.

## Materials and methods

### Materials and biochar characterization

The cationic Basic Red 46 dye was purchased from Shanghai Macklin Biochemical Co., Ltd. The bamboo and rice straw biomass wastes were obtained from a bamboo forest and cropland, respectively, in Hangzhou, Zhejiang province, China. The biomass was air-dried for 48 h and grounded through 100 mesh sieves. The dry biomass was compacted in a ceramic pot and pyrolyzed under oxygen-limited conditions for 6 h at a temperature of 500°C. The heating rate was regulated at 10°C min^-1^ to achieve the desired temperature. The obtained biochars were grounded and passed through 100 mesh sieves before use. The final samples were named B500 for Bamboo and R500 for rice straw, respectively.

Biochar surface morphology was observed by scanning electron microscopy (SEM; Phenom ProX, Philip, Holland). The surface area (S_BET_) and pore structure parameters of biochar samples were measured by the N_2_/BET method (ASAP 2020 Plus, Micromeritics, USA). Functional groups on the biochar surfaces were analyzed by Fourier transform infrared (FTIR) spectroscopy (VERTEX 70, Bruker, Germany). The synthesized samples were subjected to X-ray diffraction by a diffractometer (XRD, Philips Analytical, PW-3040).

### Adsorption experiments

The BR46 adsorption experiments by biochars were executed in batch mode. A stock solution of BR46 (30 mg/L) was prepared with ultra-pure water for the adsorption kinetics. Experiments to investigate the adsorption kinetics of BR46 onto biochar were performed in series. A 50 ml centrifuge tube was filled with 0.02g of biochar, and 20 ml of BR46 dye solution was added to it. The samples were shaken in a horizontal shaker (DHZ-DA, large capacity full temperature oscillator, China) at 200 rpm at 25°C for 5min, 10min, 0.5, 1,2,4,6,10,16,24,36, and 48 h. After shaking the samples, they were centrifuged (TGL-15B, Anke, China) at 5000 rpm for 10 minutes and the supernatant filtered through a 0.45μm PES filter. The remanent BR46 solution was measured using a UV-VIS spectrophotometer (DR 6000, Hach, USA) and operating at a wavelength of 531 nm. The large capacity full temperature oscillator (DHZ-DA) was used to maintain the experimental temperature.

Adsorption isotherm experiments were conducted with initial concentrations from 5 to 60 mg/L at 15, 25, and 35°C. A 50 ml centrifuge tube was filled with 0.02g of biochar, and 20 ml of BR46 dye solution was added to it. The samples were kept in a horizontal shaker and shaken at 200 rpm. This process was performed at 15, 25, and 35°C for all samples, respectively until adsorption equilibrium was attained.

The concentration of the aqueous BR46 solution was ascertained using the corresponding techniques above. Another series of batch experiments investigated the effects of the solution pH on the adsorption of BR46 by biochar. The pH of the BR46 solution ranged between 3 and 11, and the adsorption process was similar to the above experimental procedures.

In each experiment, control samples (i.e., samples without biochar or BR46) were concurrently prepared. However, during the control experiment, no significant improvement was observed for concentrations of the BR46. Meanwhile, all tests were repeated three times to obtain the mean value.

### Data analysis

#### Adsorption kinetic models

In the experiment on adsorption kinetics, the amount of BR46 adsorbed was calculated at different time intervals as follows:

$$q_{t}=\frac{(C_{0}-C_{t})V}{m}$$ (1)

Where *C*_*0*_ is the initial concentration of BR46 (mg/L); *C*_*t*_ is the concentration of BR46 at time t (mg/L); *V* is the volume of the solution (L), and m is the mass of the biochar used (g).

In this study, the pseudo-first-order and the pseudo-second-order models were used to evaluate the experimental data. Two models are shown in Eqs (2) and (3), respectively.


$$\ln(q_{e}-q_{t})=\ln\;q_{e}-k_{1}t$$ (2)

$$\frac{t}{q_{t}}=\frac{1}{k_{2}q_{e}^{2}}+\frac{t}{q_{e}}$$ (3)

Where *q*_*e*_ (mg/g) are the amounts of adsorbed adsorbate at equilibrium; *k*_*1*_ (h^-1^) is the rate constant of pseudo-first-order model; *k*_*2*_ (mg/(g·h)) is the reaction rate constants of the pseudo-second-order model.

#### Isotherm models

Isotherm adsorption data were fitted by Langmuir model (4) and Freundlich model (5). Langmuir model is valid for single layer adsorption coverage of each molecule on an entirely homogeneous surface. However, Freundlich isotherm can also be applied to non-ideal adsorption on heterogeneous surfaces and multilayer sorption.


$$\frac{1}{q_{e}}=\frac{1}{q_{m}K_{l}C_{e}}+\frac{1}{q_{m}}$$ (4)

$$\log q_{e}=\log\;K_{f}+\frac{1}{n}\log\;C_{e}$$ (5)

Where *C*_*e*_ (mg/L) is the BR46 concentration in the solution phase at the equilibrium time; The constant *K*_*l*_ (L/g) is the Langmuir equilibrium constant, and *q*_*m*_ (mg/g) is the maximum adsorption capacity; *K*_*f*_ (mg/g) is the Freundlich constant represented by the sorption and *n* is the Freundlich exponent.

#### Thermodynamic analysis

Specific critical thermodynamic parameters must be considered to evaluate any adsorption process [45] properly. Standard enthalpy (ΔH, kJ/mol), standard entropy (ΔS, J/mol K), and Standard Gibbs free energy (ΔG, kJ/mol) are critical parameters of thermodynamics that must be considered for proper evaluation of the whole adsorption process [12, 46]. Here, ΔG is calculated by K_d,_ which comes from q_e_ over C_e_. The values of ΔG, ΔH, and ΔS derived from equations as follows:

$$\Delta G=-RT\;\ln\;K_{d}$$ (6)

ΔG=ΔH−TΔS (7)

$$\ln\;K_{d}=-\Delta H/RT+\Delta S/R$$ (8)

Where *R* is the universal gas constant (8.314 J/(mol·K)); *T* is the absolute temperature (K), and K_d_ is the distribution coefficient (L/kg).

## Results and discussion

### Biochar characterization

The specific surface area, pore volume, and diameter of B500 and R500 shown in Table 1 change for each biochar, representing the internal solid structure model. A porous biochar structure is essential to determining the adsorption properties, but the adsorbent surface chemistry should not be overlooked [47]. The approximate surface area of B500 and R500 was found to be 1.99 m^2^/g and 5.21 m^2^/g. The average pore volume is 0.004 cm^3^/g for B500 and 0.012 cm^3^/g for R500. However, it is widely agreed that by conventional IUPAC (1972) classification, pores in diameter were divided into micropores (< 2 nm), mesopores (2–50 nm), and macropores (> 50 nm) [48]. The average pore size of the adsorbents used was 7.71 nm and 9.32 nm, respectively, and are structurally mesoporous [49].

**Table 1:** Pore structure of biochars by the N_2_/BET method.

Biochar	Specific surface area (m^2^/g)	Pore volume (cm^3^/g)	Average pore size(nm)
B500	1.99	0.004	7.71
R500	5.21	0.012	9.32

**Fig 1:**
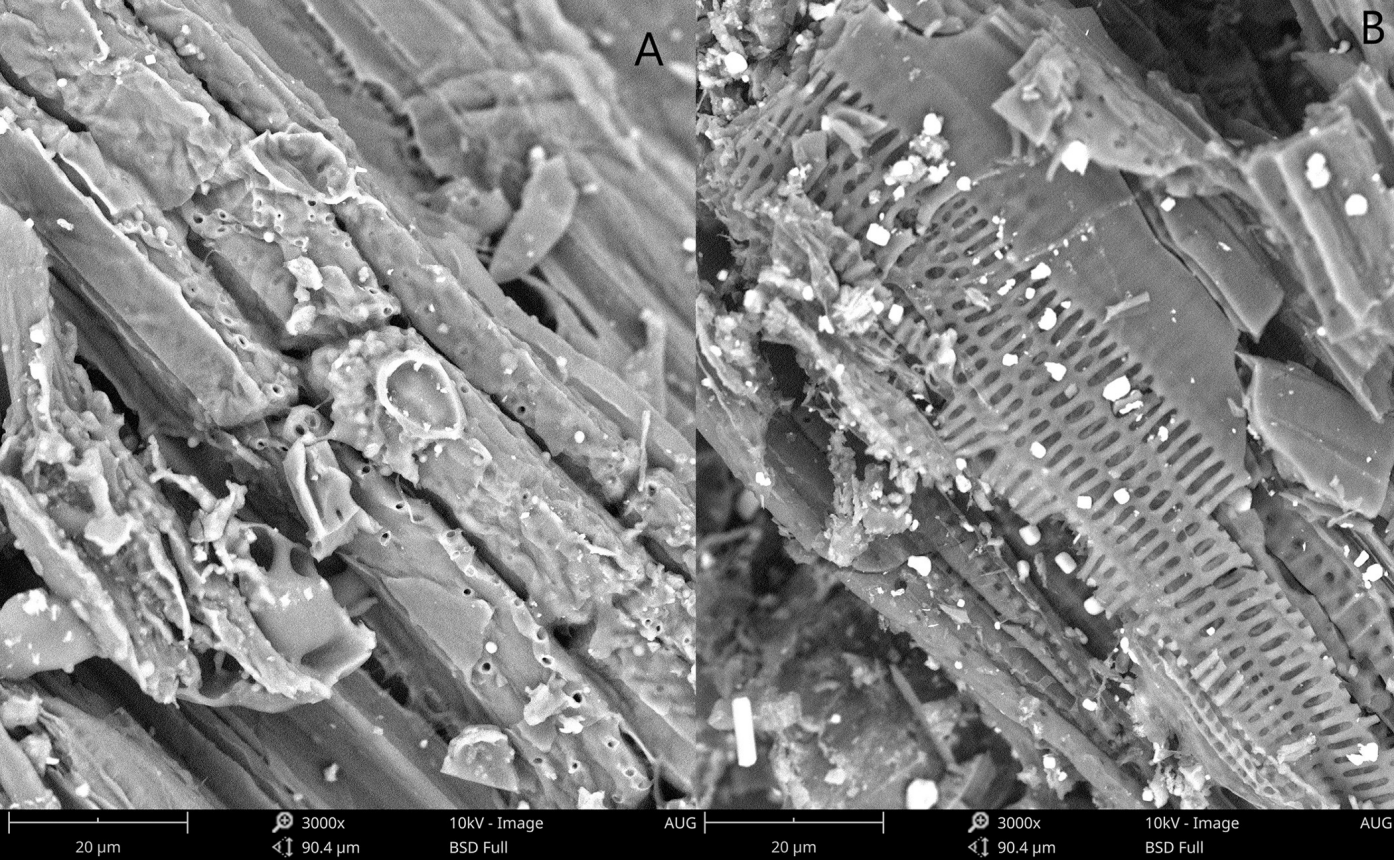
SEM images of B500 (A) and R500 (B) (×3000).

The pores of the adsorbents are visible from the SEM image in (Fig 1A and 1B) and showed the detailed surface characteristics of the adsorbent. The surface of the B500 was thick and had a smaller pore volume and size (Fig 1A), while the surface of the R500 (Fig 1B) was thin and had a larger pore volume and size. Compared to B500, the pore of R500 was more prominent than that of B500, which is consistent with the results of Table 1.

Rice straw biochar (Fig 1B) is a porous material composed of irregular plates with a broad, accessible surface area that provides efficient adsorption sites [50]. The surface of the bamboo biochar sample looked to be rougher and had a lower surface area than the rice straw biochar sample. B500 and R500’s mesoporous shape facilitates dye adsorption [3]. R500 had numerous minor pathways, which might have resulted from the pyrolysis process or property of the raw material. Overall, rice straw biochar had a higher porosity than bamboo biochar.

**Fig 2:**
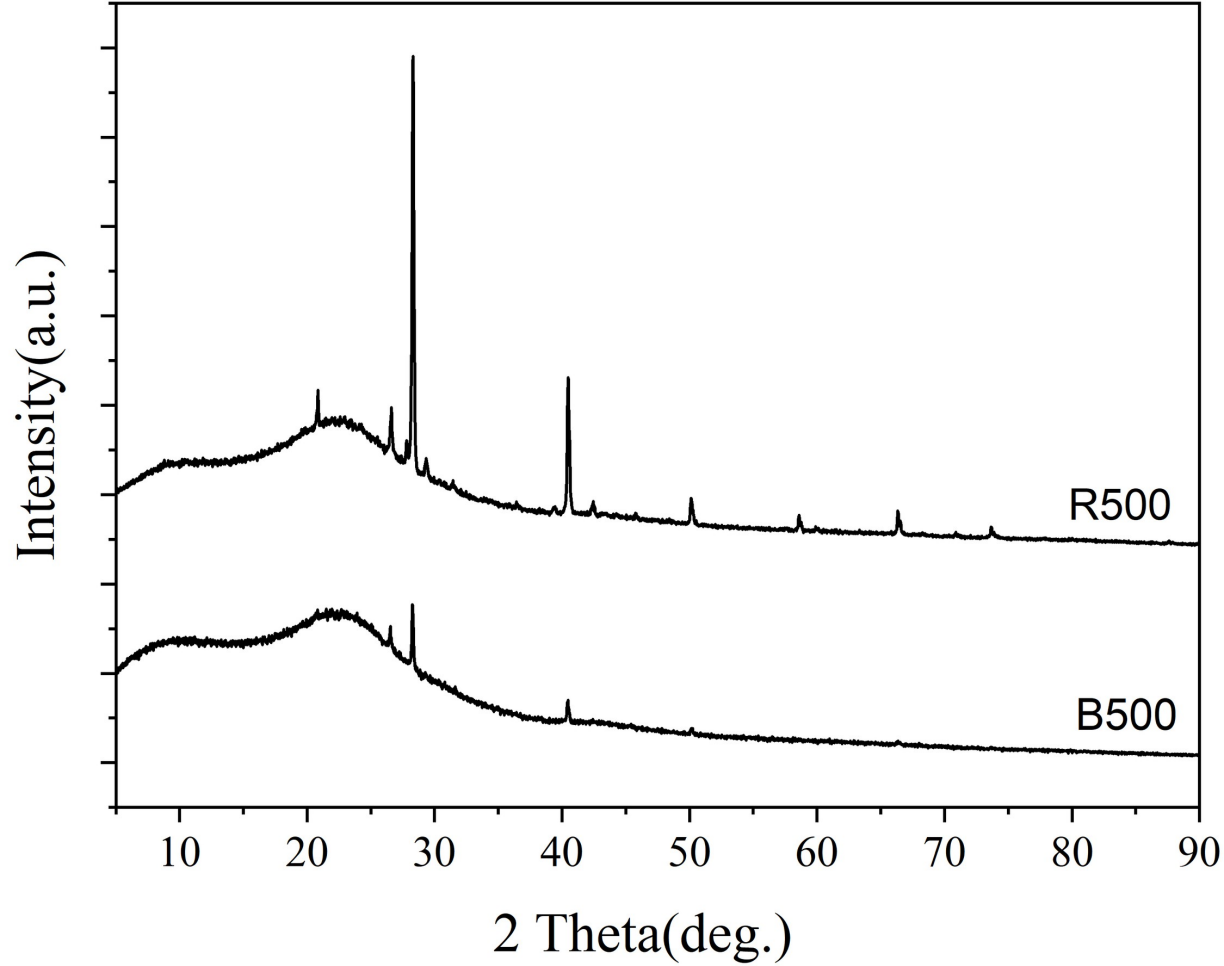
X-ray diffraction patterns of bamboo and rice straw biochar produced at 500°C.

XRD analysis was used to evaluate the degree to which the samples were crystalline or amorphous. The XRD patterns of samples are presented in (Fig 2). The samples were analyzed in the region of 2 Theta (θ) and angle range of 0˚- 90˚ at room temperature. XRD patterns of bamboo and rice straw biochars (Fig 2) indicated the presence of inorganic minerals such as SiO_2_ (quartz, 2θ = 20.86°, 26.62°), KCl (sylvite, 2θ = 28.31°, 40.50°), and CaCO_3_ (calcite, 2θ = 50.58°) [51]. When a substance is crystalline, well-defined peaks can be observed, whereas non-crystalline or amorphous materials exhibit hollow peaks [52]. Additionally, a highly sharp and strong peak shows that the material has developed a pure crystalline structure due to the elimination of hemicellulose and lignin [53]. All biochars had similar diffractogram patterns. Concluding, KCl was a dominant crystalline phase with a small amount of SiO_2_ and CaCO_3_. Bamboo and rice straw are amorphous due to the presence of hemicellulose, lignin, and other comparable components [18]. These findings corroborate those of Manna et al. [50].

**Fig 3:**
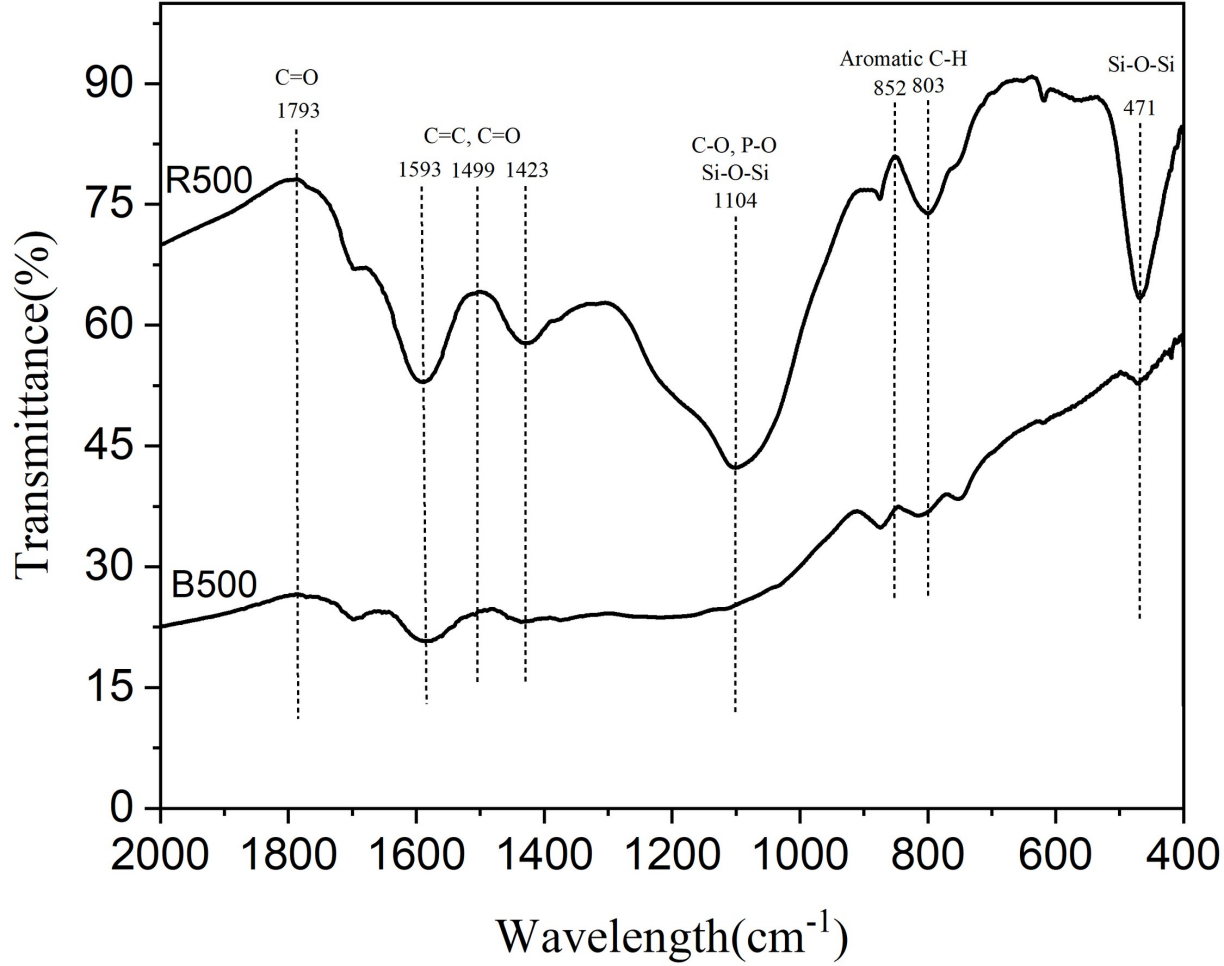
Fourier Transform Infrared (FTIR) spectroscopic analysis of biochar.

Fig 3 shows the FTIR spectra of B500 and R500. The fundamental functional groups on the adsorbent surface were determined using FTIR analysis to identify possible interactions between the dye molecules and the adsorbent’s functional groups. From (Fig 3), peaks observed at 1793 cm^-1^ indicate the presence of the (C = O) stretching group [54]. The peak intensities from (1593 to1423 cm^-1^) indicate the stretching of aromatic components (C = C) and, to a smaller extent (C = O), stretching of conjugated ketones and quinones [51]. The bands around 1000–1100 cm^-1^ were associated with the Si-O-Si group, the P-O bond of phosphate and the C-O bond of carbonate[ref]. Sharp peaks shown at (876–802 cm^-1^) indicate the stretching vibrations of aromatic C-H group [53]. The Si-O-Si group is responsible for adsorption in the 471 cm^-1^ range [54]. In comparison, R500 biochar confirmed the high Si content since a band was observed at 471 cm^−1^, which is commonly attributed to Si content [54]. The presence of cellulose, hemicelluloses, and lignin is almost certainly what caused the formation of these bands. In addition, agricultural biomasses primarily consist of lignin, cellulose, hemicelluloses, and proteins, making them excellent dye adsorbents [55].

### Adsorption kinetics

**Fig 4:**
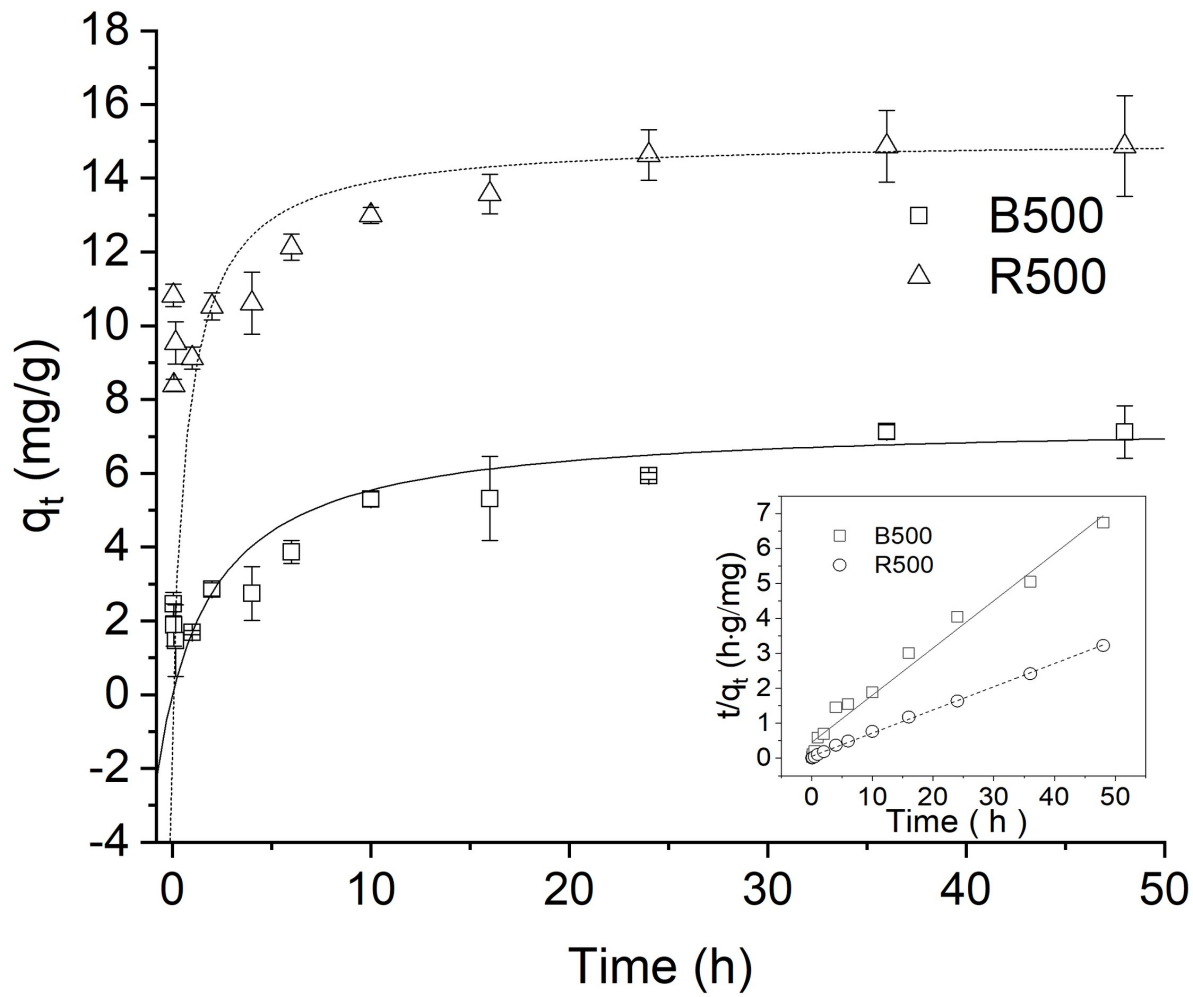
Adsorption kinetics (pseudo-second-order model) of BR46 on B500 and R500 (pH = 7.0). The insert is the linear plot of the pseudo-second-order model fit. Error bars indicate the SD.

The dye adsorption behavior was found to be time-dependent in this analysis. This was observed by adjusting the equilibrium time between adsorbate and adsorbent in the range of 5min-48 h [56]. The adsorption capacity of the dye as a function of contact time, as shown in (Fig 4), indicates that the equilibriums between the dye and the biochars were almost reached at 24 h for B500 and R500 biochars. The equilibrium between the dye and the biochars is comparable to what has been observed previously [44, 57]. A fast adsorption process can be observed during the beginning of adsorption, and adsorption capacity of 54.4% and 81.5% at 6h for B500 and R500, respectively.

After 6 h, adsorption capacity increased slowly, and adsorption almost reached equilibrium at 24 h. However, different results have been reported for equilibrium studies on the adsorption of Basic Yellow 28 and Basic Red 46 by boron industry waste as compared to the adsorbents used in this study [8]. Additionally, equilibrium was achieved after 10 h for the removal of Reactive Red 195 dye using chitosan-coacervated particles as compared to the adsorbent used in this study [58]. At the equilibrium time, the adsorption capacity of 7.12 mg/g and 14.87 mg/g for B500 and R500 were obtained, respectively. Clearly, R500 was more effective in adsorbing BR46 than B500.

The data from the adsorption experiments were fitted by kinetic models, including pseudo-first-order and pseudo-second-order models. The experimental data and model calculated values are identified with the correlation coefficient (R^2^) in Table 2. The pseudo-first-order model does not match the experimental data for the BR46 adsorption onto biochar (R^2^<0.89). The low R^2^ values and the disparity between the measured experimental equilibrium sorption indicate that the pseudo-first-order was unable to describe the adsorption kinetics [29]. On the other hand, experimental results showed strong agreement with the pseudo-second-order models equation with R^2^>0.99, which illustrates the compliance between experimental and calculated equilibrium sorption for the pseudo-second-order.

**Table 2:** Kinetic parameters for the adsorption of BR46 onto biochars.

Biochars	Pseudo-first-order			Pseudo-second-order		
	q_e_ (mg/g)	k_1_ (h^-1^)	R^2^	q_e_(mg/g)	k_2_(mg/(g·h))	R^2^
B500	3.33	0.144	0.845	7.04	0.0402	0.996
R500	4.62	0.089	0.532	15.06	0.0787	0.998

Thus, chemisorption involving π-π interactions, electrostatic interactions, and chemical reactions between biochar surface functions and dye molecules may be used to modulate the sorption process of cationic dyes on both biochars. Similar findings have been made with dye adsorption onto various forms of biochars [21]. Corresponding values of q_e_ can also be found in the experimental data and model calculations.

### Influence of pH

**Fig 5:**
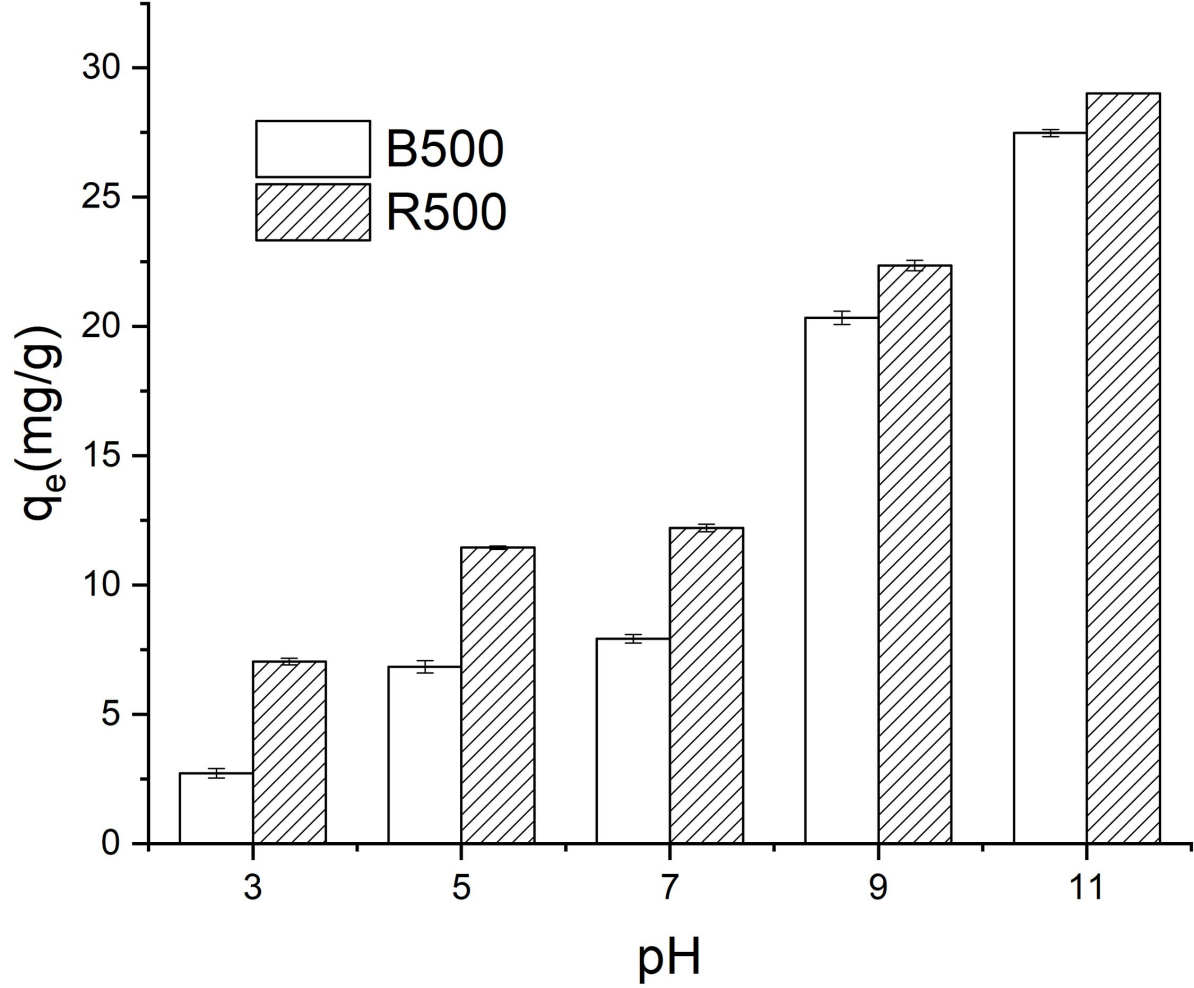
Effect of pH on adsorption capacity of Basic Red 46 on B500 and R500.

The pH is a significant control parameter in dye adsorption. Its effect on BR46 adsorption on biochar is studied over the pH range of 3–11, with the results shown in (Fig 5). It was observed that with the subsequent increase in pH, the adsorption capacity begins to increase. This result is consistent with the previous studies [59, 60]. Compared to B500, the adsorption capacities of BR46 on R500 were all much higher. In the range of 3–11 pH values, the adsorption capacity of BR46 on B500 ranged from 2.73 mg/g to 27.48 mg/g, and for R500, the values varied from 7.05 mg/g to 29.01 mg/g. Obviously, low pH does not strongly influence the adsorption process of BR46 on B500 and R500. In the pH range of < 7, the adsorption capacity increased steadily, and the adsorbed quantity of BR46 increased sharply for pH values between 9 and 11.

There are isoelectric points for biochar, and the surface charges of biochar will change during the different pH values. The isoelectric points of biochar in this study reported were between 3 and 5, which is in good accordance with the study as stated for the effectiveness and mechanisms of dye adsorption of straw-based biochar and sorption of lincomycin by manure-derived biochars from water [61, 62]. On the other side, BR46 is a cationic dye, and then the interaction between dye and biochar changed with the variation of pH values. The positive charge of biochar and cationic dye resulted in electrostatic repulsion during low pH, and the adsorption capacity was relatively low. At high pH, the positive charge of BR46 can be adsorbed by the negative charge of biochar mostly through electrostatic interaction, resulting in a significant increase in adsorption capacity. The results on pH patterns on dye sorption are consistent with the study as reported for removal of malachite green from aqueous solution, removal of congo red dye from aqueous solutions, and also removal of C. I Basic Red 9 from aqueous solution [12, 26, 63].

### Adsorption isotherms

**Fig 6:**
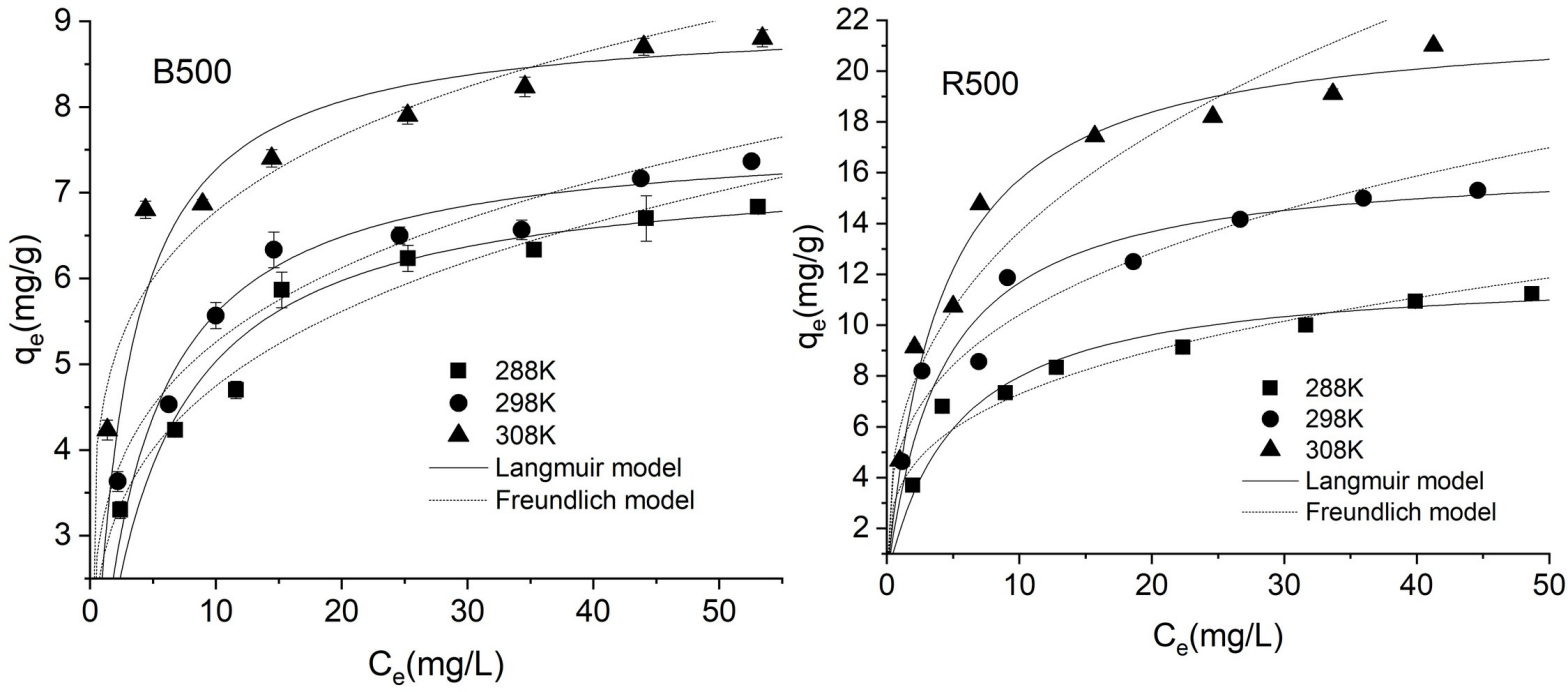
Adsorption isotherms for the adsorption of BR46 on B500 and R500 (pH = 7.0).

Equilibrium isotherm modeling is required to determine the distribution of dye molecules on the biochar surface and to comprehend the dye-biochar adsorption mechanism. Langmuir and Freundlich isotherm model equations were used to analyze the adsorption equilibrium data for all biochar samples at different temperatures. Fig 6 showed that the adsorption isotherms of BR46 on bamboo biomass-derived biochar and rice straw-derived biochar. With the increase of adsorption temperature, the adsorption capacities of BR46 on both B500 and R500 increased. Especially for R500, adsorption capacity increased markedly. The most considerable capacities of 8.80 mg/g and 21.00 mg/g for B500 and R500 were obtained at 308K, respectively.

The isotherm parameters of the Langmuir and Freundlich equation are summarized in Table 3. The adsorption isotherm results from Table 3 showed that the Langmuir model fitted the data well (R^2^>0.99) than the Freundlich model (R^2^ = 0.898–0.958) for all biochars at different temperatures. These findings suggested that the adsorbent adsorption sites of BR46 were homogeneous, consistent with the assumption of the Langmuir model [29]. In contrast to the Freundlich equation, the Langmuir equation generally better represents the isotherm data for all biochars at different temperatures. Previous studies also showed better fittings of the Langmuir equations than the Freundlich equations to isotherm data of BR46 sorption onto adsorbents [64, 65]. In this study, the values of q_max_ increased with the increase of temperature, which implied that the adsorption easily occurs at high temperatures. The q_max_ values of 7.35–9.06 mg/g for B500 and 12.14–22.12 mg/g for R500 were observed in the temperature range of 288K-308K. The maximum adsorption capacity values (q_max_) for the removal of different dyes based on agricultural waste, fruit/fish waste, etc., have been summarized in Table 4. From the data in Table 4, it can be determined that the rice straw biochar used in the present study serves as an adsorbent for basic dye. BR46 has a considerable q_max_ value of 22.12 mg/g relative to q_max_ values for other fruit/vegetable waste and agricultural waste for the removal of different dyes. As a result, the essential q_max_ value recorded in the present study demonstrated the potential suitability of rice straw as an innovative adsorbent for the removal of BR46. It appears, therefore, that the availability of this material and its ability to eliminate cationic dyes are advantageous and encouraging for the safety of the environment.

**Table 3:** Langmuir and Freundlich models parameters for BR46 adsorption on biochars.

Biochars	T(K)	Langmuir model			Freundlich model		
		*q*_*max*_(mg/g)	*k*_*l*_ (L/mg)	R^2^	*k*_*f*_(mg/g)	1/n	R^2^
B500	288	7.35	0.218	0.996	2.71	0.243	0.958
	298	7.72	0.264	0.996	3.17	0.220	0.951
	308	9.06	0.407	0.998	4.51	0.177	0.898
R500	288	12.14	0.191	0.994	3.63	0.303	0.912
	298	16.53	0.240	0.994	5.16	0.305	0.924
	308	22.12	0.246	0.996	5.98	0.359	0.936

**Table 4:** Comparing the adsorption capacities of adsorbents obtained from agricultural waste and fruit/vegetable waste for separate dye removal.

Adsorbents	Adsorption capacity (mg/g)	Reference
Rice straw	22.12	Present study
Cereal Chaff	20.30	[66]
Orange Peel	18.6	[67]
Wheat Shells	16.56	[68]
Raw green nanoceria	16.39	[69]
Canola stalk	13.22	[60]
Banana peel	3.88	[24]
Coarse grinded what straw	3.82	[70]
Fine grinded wheat straw	2.23	[70]

The K_L_ value represented Langmuir constant which relates to the binding energy of adsorption. From Table 3, the K_L_ value increased with the increase of temperature for both biochars, which indicated that high temperature is favorable for adsorption of BR46 on B500 and R500.

Numerous mechanisms, including π-π interaction, electrostatic interactions, cation exchange, complexation, precipitation, and chemical reduction, may be involved in the adsorption of BR46 onto various adsorbents [21]. In this study, electrostatic interaction (cation and anion attraction) is an important mechanism in the adsorption of BR46 onto biochar at experimental conditions, as described in the influence of pH section above. O-containing functional groups are considered to serve as H-bond acceptors [7, 55]. The FTIR analysis performed in this study revealed that R500 contains more O-containing functional groups than B500. R500 had a greater capacity for adsorption than B500, implying that another mechanism is most likely H-bond interaction [54]. This result is also reported by other studies [7, 54]. Additionally, some studies suggested that the structure of biochar acts as a π -electron-donor[7, 58]. In this study, BR46 can act as an electron-acceptor, contributing to n-π EDA (Electron Donor Acceptor) interaction.

### Thermodynamic analysis

**Fig 7:**
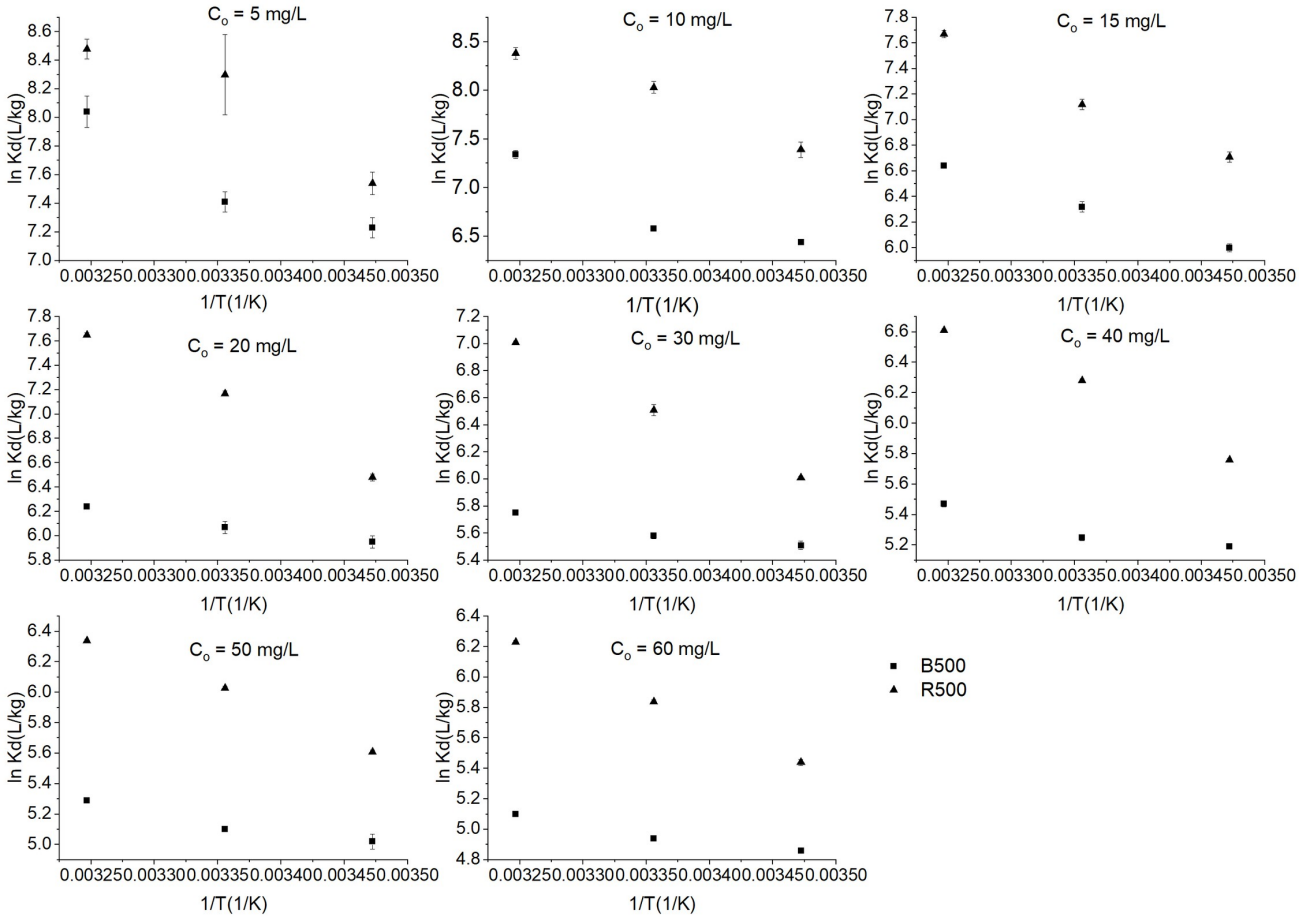
Effect of temperature on sorption coefficient (K_d_) for Basic Red 46 on biochars.

Eqs (6)–(8) were used to measure three thermodynamic parameters, including the typical Gibbs free energy (ΔG, kJ/mol), enthalpy (ΔH, kJ/mol), and entropy (ΔS, J/mol·K) to assess the effect of temperature on adsorption processes [63]. The relations between lnK_d_ and 1/T for BR46 adsorption on different biochars were shown in (Fig 7), and well linear fitting (R^2^>0.99) of lnK_d_ and 1/T was obtained. The ΔG values were calculated from lnK_d_ [26], then ΔS and ΔH are calculated from the Eq (8). Table 5 showed thermodynamic parameters for the adsorption of BR46 onto biochar. The ΔG values for all the samples were negative, which indicates that the adsorption of BR46 onto biochar was thermodynamically feasible and spontaneous [44]. With the increase of temperature, the absolute values of the ΔG for B500 and R500 increased. This result indicated that high temperature is favorable for adsorption. Compared to R500, the absolute values of the ΔG of B500 were relatively low, which suggested that the adsorption process was less thermodynamically feasible when the bamboo biomass biochar was used [71].

**Table 5:** Thermodynamic parameters for the adsorption of BR46 onto biochar.

Biochar	Temperature (K)	Thermodynamic Parameters		
		ΔG (kJ/mol)	ΔH (kJ/mol)	ΔS (J/(mol·K))
B500	288	-11.63–17.30	8.74–33.08	71.73–167.54
	298	-11.83–17.74		
	308	-12.22–19.25		
R500	288	-13.03–18.05	26.87–43.25	140.09–202.27
	298	-13.98–19.84		
	308	-14.92–20.31		

ΔH is used to distinguish the nature of the adsorption [58]. The ΔH values for the biochar ranged from 8.74–43.25 kJ/mol from Table 5, indicating that the adsorption process was endothermic and more feasible at higher temperatures. It was observed that there were higher values of ΔH for the rice straw biochar than that of the bamboo biomass biochar [72]. When the ΔS has a positive value, this implies an increase of adsorbate molecules on the solid surface than in the solution [73]. The ΔS values for the biochar were positive, ranging from 71.73–202.27 J/(mol·K), as shown in Table 5. This indicates an increase in the randomness BR46-biochar interface during the adsorption process [4].

### Adsorption affinity

**Fig 8:**
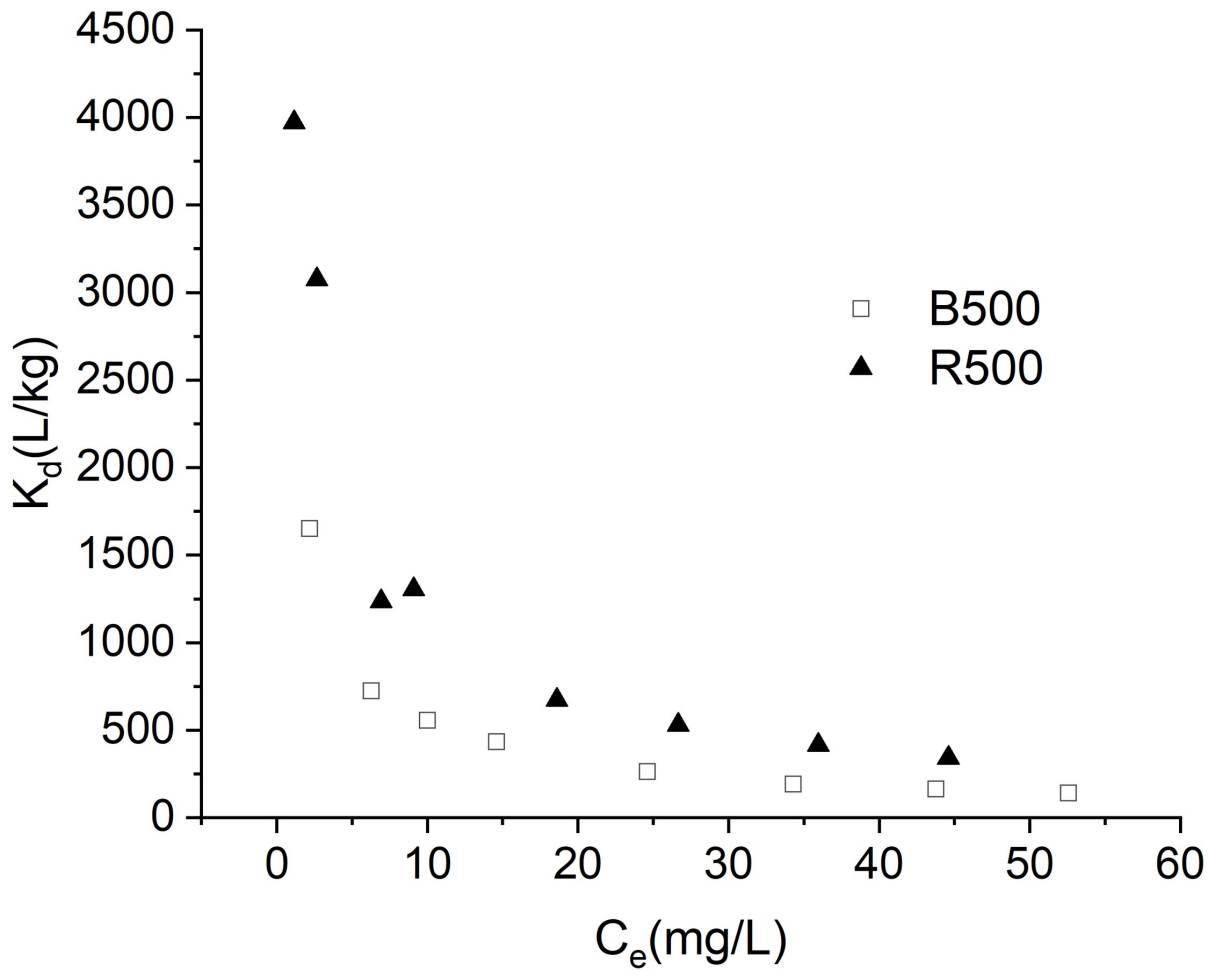
Adsorption coefficient (K_d_) values for sorption of Basic Red 46 on biochar.

K_d_ represents the adsorption affinity of absorbents, and the K_d_ values are shown in (Fig 8) for both biochar forms at BR46 experimental concentration. Larger K_d_ values 3971.41 L/kg, consistent with the results calculated from the Langmuir model, have been found for rice straw biochar. The K_d_ values of B500 were only in the range of 140–1660 L/kg. It was observed that R500 has a comparatively high affinity for BR46 compared to B500, particularly at low BR 46 concentrations. In general, the K_d_ values were in the order and ranged from 10^2^ to 10^3^ for B500 and R500, and similar results were reported by Wang et al. [44]. Additionally, as the BR46 concentration increased, the adsorption coefficients decreased. These results suggested that the adsorption affinity of BR46 was dependent on concentration and was higher at lower levels.

## Conclusion

This work demonstrated that rice straw biochar has excellent surface characteristics and is an efficient adsorbent to remove BR46 from aqueous solutions. However, the dye adsorption efficiency of bamboo biochar was relatively poor. The resulting biochars demonstrated superior surface properties and better adsorptive efficiency when compared to other similar adsorbents. For B500 and R500, the adsorption equilibrium was determined after 24 h, and the second-pseudo-order model well fit the experimental data. The results indicated that the pH of the solution had a substantial effect on the adsorption processes and that adsorption capacity increased with increasing pH, reaching a maximum at pH 9 and 11. The maximum sorption capacity (q_max_) of B500 and R500 was 9.058 and 22.124 mg/g, respectively. The Langmuir model predicted the adsorption of BR46 on B500 and R500 more accurately than the Freundlich model. The sorption mechanism of BR46 onto biochar-derived bamboo and rice straw biomass is influenced by physical and chemical interactions, including surface adsorption, π-π interaction, chemical interaction, and electrostatic interaction. According to the thermodynamic study, the adsorption process was spontaneous and endothermic. R500 has a more significant adsorption coefficient (K_d_) than B500. K_d_ values were in the range of 10^2^−10^3^ for two biochars. Overall, direct pyrolysis of rice straw waste provides a viable and cost-efficient method for transforming a large volume of waste into a suitable adsorbent for successful dye-contaminated water and wastewater treatment.

## Supporting information

S1 Table Adsorption kinetics for the adsorption of BR46 onto biochars. (XLSX)

S2 Table Adsorption isotherm of BR46 adsorption onto biochars. (XLSX)

S3 Table Effect of pH on BR46 adsorption on biochars. (XLSX)

S4 Table Adsorption coefficient (K_d_) values for sorption of Basic Red 46 on biochar. (XLSX)
